# Phosphate, Calcification in Blood, and Mineral Stress: The Physiologic Blood Mineral Buffering System and Its Association with Cardiovascular Risk

**DOI:** 10.1155/2018/9182078

**Published:** 2018-09-02

**Authors:** Andreas Pasch, Willi Jahnen-Dechent, Edward R. Smith

**Affiliations:** ^1^Department of Biomedical Research, University of Bern, 3008 Bern, Switzerland; ^2^Calciscon AG, 2560 Nidau, Switzerland; ^3^Biointerface Laboratory, RWTH Aachen University Hospital, 52074 Aachen, Germany; ^4^Department of Nephrology, The Royal Melbourne Hospital, Melbourne, Victoria, Australia; ^5^Department of Medicine, Royal Melbourne Hospital, University of Melbourne, Melbourne, Victoria, Australia

## Abstract

Phosphate is an important cardiovascular risk factor and lowering elevated blood phosphate concentrations is a main therapeutic target in kidney patients. Phosphate is subject to the* blood mineral buffering system* which controls the precipitation of calcium and phosphate. Calciprotein particles (CPP), self-assembling complexes of calcium phosphate and serum proteins, are the nanomorphological correlates of this system. CPP1 are spherical, 50-100 nm in diameter, and contain amorphous mineral. CPP2 are oblongated, 100-200nm in the long axis, and they contain a crystalline mineral core. The relative abundance and biological activity of these particles are a matter of intense research, because they can cause oxidative stress, inflammation, and calcification in cellular assay. Therapeutically reducing this endogenous stressor by prolonging* crystal formation time* might improve patient outcome. This concise review article summarizes our current knowledge about the* blood mineral buffering system* and proposes* Mineral Stress* as a novel modifiable cardiovascular risk factor. It furthermore outlines possible implications this might have for improving patient care.

## 1. Cardiovascular Disease Is a Major Complication of Chronic Kidney Disease

Patients suffering from chronic kidney disease (CKD), and even more so those with advanced disease already undergoing dialysis treatment, are at very high risk of cardiovascular morbidity and mortality [1].

The realization that traditional major cardiovascular risk factors like hypertension, diabetes, smoking, and high cholesterol do not fully explain this observation suggested the involvement of nontraditional, uremia-related risk factors and spawned considerable research efforts over the last decades. Vascular calcification, oxidative stress, smoldering inflammation, and malnutrition are commonplace in uremia [2, 3] and have been identified as significant correlates of the increased risk in this setting. However, the direction of causality for these relationships largely remains unresolved. This is especially true for vascular calcification, where it is uncertain whether it is the functional or structural impact of calcium phosphate deposition (e.g., vessel stiffness, altered remodeling, and plaque instability) or the underlying processes leading to ectopic mineralization that are injurious and more directly related to outcome. Regardless, the prevailing dogma attributes many of these manifestations to disturbances of mineral metabolism, mostly notably blood phosphate concentration, and the interconnected bone-vascular axis.

Blood phosphate was identified by Block and colleagues as a major nontraditional cardiovascular risk factor in patients with kidney disease [4, 5]. This major breakthrough in our understanding led to the institution and subsequent intensification of therapies aimed at lowering blood phosphate level. Unfortunately, despite often aggressive attempts to address this important therapeutic target, concomitant reductions in cardiovascular prognosis have not been achieved. This raises questions about our understanding of the pathophysiologic mechanism(s) underlying cardiovascular disease in kidney disease patients and whether the assumptions on which they are based are indeed correct.

## 2. Current Concepts Are Centered around Phosphate

Improvements in patient treatment are usually driven by the identification of new therapeutic targets by academic research followed by the provision of diagnostic and therapeutic products by industry. Here, the discovery of the importance of phosphate and compelling data showing profound effects in animals fed phosphate-rich diets [6], led to the promotion and wide-spread use of calcium-containing and, more recently, calcium-free phosphate binders.

While no prospective randomized controlled trial has demonstrated the favorable effects of phosphate lowering on outcome, numerous observational studies have demonstrated an association between phosphate concentrations and cardiovascular events [7]. While these associations appear robust when large patient cohorts are considered, it is much less clear what the ideal phosphate concentration is for the individual patient.

Nonetheless, phosphate is currently regarded as a major player in the syndrome of Chronic Kidney Disease-Mineral and Bone Disorder (CKD-MBD), which assigns clinical significance and a common pathophysiologic basis to the combination of vascular calcifications, bone abnormalities, and derangements in laboratory values of various mineral metabolites. Phosphate is regarded as the main mediator of outcome and is embedded into its complex physiological network of hormonal regulators. Nutrition (diet) and efflux from bone are considered the main sources of circulating phosphate. Complementing phosphate binder therapy and dietary interventions, hyperparathyroidism and high bone turnover have subsequently become additional treatment targets, in part with a view to controlling unwanted phosphate flux from bone.

Despite considerable efforts in recent years and the emergence of new players like FGF23, CKD-MBD is far from well understood. Our pathophysiologic concepts are incomplete and unfortunately supported only by moderate evidence at best. This makes CKD-MBD difficult to understand, even for the experts, and complex to treat for the clinical practitioners with limited therapeutic options. Thus, while lowering phosphate concentrations is of importance for kidney disease patients, it is not yet clear how elevated phosphate levels translate into adverse outcome.

## 3. Phosphate as Part of the* Blood Mineral Buffering System*

The recognition that calcium and phosphate precipitate to form ectopic depositions led to the relatively crude construct of the calcium phosphate product. However, this was found to offer little information over individual component measurements and does not reflect the fundamentally more complex physiochemical aspects of biological mineralization. A promising new concept is to view phosphate in the context of a system of inhibitors or promoters of calcium phosphate crystallization. This crystallization process progresses through a series of steps towards the end product carbonated hydroxyapatite. First, small amorphous ion clusters of calcium phosphate, so-called Posner clusters, are formed. These then transform to octacalcium phosphate and finally to hydroxyapatite. This natural process of mineral ripening also occurs in maturing bone [8]. While this process progresses rapidly in simple aqueous solution, it is much delayed by protein and non-protein components in complex biological fluids like blood. One mechanism of crystal growth inhibition is the sequestration of the initial Posner ion clusters by growth-inhibiting proteins. The liver-derived plasma protein fetuin-A is a major regulator of mineralization. Fetuin-A inhibits the growth of small calcium phosphate crystal nuclei through a molecular shielding mechanism mediated by its amino-terminal cystatin-like domain [9].

Upon strong supersaturation of blood with calcium and phosphate, mineral-laden fetuin-A and other proteins self-assemble to form primary calciprotein particles (CPP1). These contain amorphous calcium phosphate and are the nanomorphological correlates of the* mineral buffering system* inherent in blood. The function of this system is to keep surplus amounts of calcium phosphate suspended until it is cleared. Besides fetuin-A and other proteins like albumin, which represent main structural components of CPP1, small molecules like magnesium, pyrophosphate, zinc, citrate, or OH^−^ influence the stability of these nanoparticles.

Over time, at least in vitro, CPP1 undergo a characteristic phase transformation into CPP2. These particles are larger and more spindle-shaped than CPP1 and contain crystalline hydroxyapatite. Importantly, the uremic environment may further condition CPP.

## 4. The* Mineral Stress* Hypothesis

The formation of CPP is a spontaneous naturally occurring process. It occurs not only in human serum, but also in fetal bovine serum-containing cell culture medium, which is rich in fetuin-A. Amorphous CPP1 exerted minor cellular responses in macrophage cell lines, while CPP2 appeared to induce oxidative stress and inflammation in macrophages [10], and oxidative stress, inflammation, and calcification in primary human aortic smooth muscle cell cultures [11, 12]. Like in any cell-based calcification assays, these results greatly depend on the amount and stability of the various CPP preparations and on the particular cell types under study, and therefore varying outcome is to be expected depending on the exact conditions of each assay. Nevertheless, it is evident that numerous studies reporting the effects of “phosphate” treatment in cell culture did indeed study effects of phosphate containing forms of CPP. Thus, the damage attributed to excess phosphate may in fact be mediated by phosphate containing more or less crystalline forms of CPP rather than by soluble phosphate. This is of crucial importance because, under this hypothesis, excessive build-up and defective clearance of CPP may be important novel cardiovascular risk factors collectively addressed as* Mineral Stress*.

**Figure 1:**
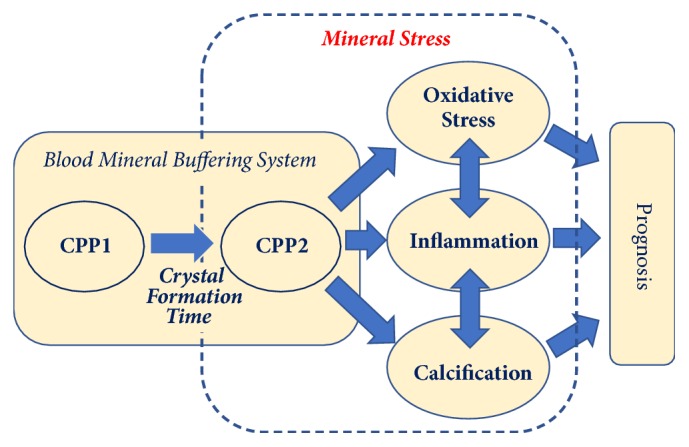
Schematic illustration of the Mineral Stress hypothesis. Mineral Stress is caused by excess calciprotein particles, which upon interaction with susceptible cells, tissues, and organs induce damage in the form of oxidative stress, inflammation, and calcification. These clinical problems then contribute to adverse outcome. CPP are formed by the blood mineral buffering system when calcium and phosphate concentrations are raised. Primary CPP (CPP1) occur earlier during excess mineral buffering, whereas CPP2 should not occur in sizeable amounts, because CPP in general are rapidly cleared from circulation. Longer crystal formation time in vitro has consistently been associated with better cardiovascular outcome in multiple clinical studies. Figure provided with courtesy from Calciscon AG.

The term* Mineral Stress* summarizes the biological consequences of the chronic exposure of cells, tissues, and organs towards circulating crystalline calcium phosphate debris, i.e., CPP (Figure 1). These include, e.g., oxidative stress, inflammation in the form of proinflammatory cytokine release, and soft tissue calcifications, which then contribute towards determining the clinical outcome.

Of note, the association of* Mineral Stress* with outcome depends not only on the magnitude of* Mineral Stress* itself, but also on the susceptibility of an individual's cells, tissues, and organs towards* Mineral Stress*.

**Figure 2:**
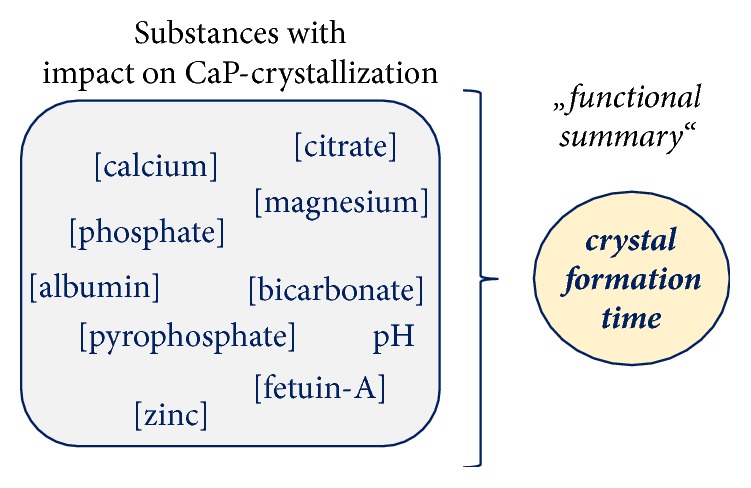
Crystal formation time is a measure of calcium phosphate crystallization. Crystal formation time functionally measures the transformation from amorphous primary (CPP1) to crystalline secondary (CPP2) calciprotein particles. It provides an integrated “functional summary” of crystallization promoting and inhibiting substances in serum and thus gives an estimate of the likelihood of CPP2-formation. Figure provided with courtesy from Calciscon AG.

*Crystal formation time*, initially invented as T_50_-time, measures the delay of CPP2-formation from CPP1* in vitro* and reflects an individual's setpoint of crystal formation in serum/blood (Figure 2). It provides a functional assessment of the* blood mineral buffering system* by integrating the interplay of the various components of this system into a single functional measurand. It thus adds important information beyond today's isolated measurement of single components which are challenging to integrate into a risk score in an objective and physiologically meaningful manner. Clinically, a more complete picture can now be seen beyond the “single puzzle pieces” seen today.

The susceptibility towards* Mineral Stress* appears to be high in kidney disease patients although the association between* crystal formation time* and excretory kidney function is largely explained by the serum levels of calcium and phosphate [13]. Thus, additional factor(s) appear to play a role which aggravate the effect of* crystal formation time* on outcome. Sedimentable fetuin-A [14] and inflammation (hsCPR and TNF-alpha) are good candidates for such additional factors [11, 14].

## 5. Clinical Implications of the* Mineral Stress* Hypothesis

Clinical studies investigating mineral-laden, sedimentable fetuin-A have associated circulating mineral debris with coronary artery calcification and with survival in chronic kidney disease. More refined scientific methods have recently become available to detect and classify naturally occurring CPP [15] and future studies using these methods will certainly provide further insight into their pathophysiological role.

As mentioned above, recently the measurement of* crystal formation time* was introduced to quantify the delay of the transformation from CPP1 to CPP2* in vitro*. This test supersaturates serum with calcium and phosphate and determines the* crystal formation time* of CPP2 formation.* Crystal formation time*, also called T_50_-time, has been associated with cardiovascular morbidity and mortality and all-cause mortality in clinical studies with chronic kidney disease stages 3 and 4 [14], kidney transplanted patients [16, 17], and hemodialysis patients [18]. Shorter* crystal formation times* have therefore been consistently associated with worse patient outcome. Further evidence lends plausibility to the importance of* crystal formation time* by demonstrating associations with the loss of transplant kidney function [17], histological changes in kidney biopsies [19], and associations with the oxygenation status of kidney tissue and vascular stiffness [20].

The therapeutic prolongation of* crystal formation time* reduces* Mineral Stress*. Interestingly,* crystal formation time* can be therapeutically modified in kidney patients [21, 22]. This opens the exciting possibility of expanding our therapeutic armamentarium by combining new therapeutic interventions aimed at* crystal formation time* instead of only focusing on isolated single components like phosphate.

One such potential therapeutic target is magnesium, which, besides phosphate, also has strong impact on the* crystal formation time* [23]. The same is likely true for citrate, zinc, and bicarbonate. These substances can be supplemented to kidney patients under supervision of their blood concentrations. Prospective clinical trials investigate the effect of dialysis (NCT03292029), citrate (NCT03565913, NTR5226), bicarbonate (NCT03301558, NCT02915601), phosphate lowering (NCT03010072), magnesium (NCT02542319, NCT02977117, NCT02621762, NCT03104166), or Vitamin K (NCT03493087) on* crystal formation time*. Such controlled studies are needed to prove the safety and effectiveness of interventions.

Redefining and expanding our conceptual focus from CKD-MBD (Mineral Bone Disorder) towards CKD-MSD (Mineral Stress Disorder) may trigger the development of new diagnostics and therapeutics.

## 6. Open Questions

It is becoming increasingly clear that* Mineral Stress* is a new cardiovascular risk factor. However, still many research questions need to be addressed. Such questions, e.g., relate to the sites of natural CPP synthesis and the distribution, clearance, and physiological role of CPP. Furthermore, detailed analyses of the pathophysiological mechanisms linking CPP with prognosis and the derived therapeutic potential when addressing this new therapeutic target are needed. Further important questions relate to the potential of CPP to promote natural and accelerated aging processes.

## 7. Summary and Outlook

In summary, naturally occurring crystalline nanoparticles and in particular the concept of CPP-mediated* Mineral Stress* will provide important new insights into the pathophysiology of cardiovascular disease. Therapeutically focusing on the* blood mineral buffering system* instead of its isolated components has the potential to improve* Mineral Stress* in CKD-patients and to lead to better outcome.
